# Arctigenin functions as a selective agonist of estrogen receptor β to restrict mTORC1 activation and consequent Th17 differentiation

**DOI:** 10.18632/oncotarget.13338

**Published:** 2016-11-14

**Authors:** Xin Wu, Bei Tong, Yan Yang, Jinque Luo, Xusheng Yuan, Zhifeng Wei, Mengfan Yue, Yufeng Xia, Yue Dai

**Affiliations:** ^1^ Department of Pharmacology of Chinese Materia Medica, China Pharmaceutical University, Tong Jia Xiang, Nanjing, China; ^2^ Institute of Pathology and Southwest Cancer Center, Southwest Hospital, Third Military Medical University, Chongqing, China

**Keywords:** arctigenin, estrogen receptor β, mTORC1 activation, Th17 cell differentiation, colitis, Immunology and Microbiology Section, Immune response, Immunity

## Abstract

Arctigenin was previously proven to inhibit Th17 cell differentiation and thereby attenuate colitis in mice by down-regulating the activation of mechanistic target of rapamycin complex 1 (mTORC1). The present study was performed to address its underlying mechanism in view of estrogen receptor (ER). The specific antagonist PHTPP or siRNA of ERβ largely diminished the inhibitory effect of arctigenin on the mTORC1 activation in T cell lines and primary CD4^+^ T cells under Th17-polarization condition, suggesting that arctigenin functioned in an ERβ-dependent manner. Moreover, arctigenin was recognized to be an agonist of ERβ, which could bind to ERβ with a moderate affinity, promote dissociation of ERβ/HSP90 complex and nuclear translocation and phosphorylation of ERβ, and increase the transcription activity. Following activation of ERβ, arctigenin inhibited the activity of mTORC1 by disruption of ERβ-raptor-mTOR complex assembly. Deficiency of ERβ markedly abolished arctigenin-mediated inhibition of Th17 cell differentiation. In colitis mice, the activation of ERβ, inhibition of mTORC1 activation and Th17 response by arctigenin were abolished by PHTPP treatment. In conclusion, ERβ might be the target protein of arctigenin responsible for inhibition of mTORC1 activation and resultant prevention of Th17 cell differentiation and colitis development.

## INTRODUCTION

Ulcerative colitis (UC) is a chronic non-specific inflammatory disease affecting the colonic mucosa, and Th17 cells have been recognized to play a crucial role in its onset and progression [1]. Recently, we reported that arctigenin, a dibenzylbutyrolactone lignan constituent of Fructus Arctii (a traditional Chinese medicine), could effectively ameliorate the colonic inflammation and crypt damage in dextran sulfate sodium (DSS)-induced colitis in mice, and it functioned mainly by inhibiting the differentiation of Th17 cells *via* down-regulation of the activation of mTORC1. Of note, the inhibitory effect of arctigenin on mTORC1 activation was independent on the classical upstream pathways of mTORC1 signaling [2, 3], and its potential target protein and underlying mechanism remained to be clarified.

Arctigenin has been categorized as phytoestrogens (mainly consisting of the isoflavonoids, coumestans and lignans derived from plants), which have structures similar to 17 β-estradiol (E2) and exert either estrogenic or anti-estrogenic activities [4, 5]. Phytoestrogens have been considered as a part of selective estrogen receptor modulators (SERMs) and a “natural” alternative to estrogen [6]. Estrogens, particularly its predominant form E2, could inhibit mTOR activation in osteoblasts [7], and phytoestrogens calycosin and liquiritigenin also inhibited the activation of mTOR pathway in breast cancer cells and glioma cells, respectively [8, 9]. These findings suggest a possibility that arctigenin acts as a ligand of ER subtype to inhibit the activation of mTORC1.

On the other hand, several studies indicated that estrogens are beneficial for the attenuation of T cell-associated diseases, such as UC, rheumatoid arthritis and experimental autoimmune encephalomyelitis (EAE) [10-12]. Estrogens partially prevented colitis in HLA-B27 transgenic model of inflammatory bowel disease, dinitrobenzene sulfonic acid, acetic acid and DSS-induced colitis model [13-16]. E2 was shown to decrease the serum level of IL-17, inhibit Th17 differentiation, and hamper disease progression in the context of EAE [11]. In ovariectomized mice, Th17 cell proportion was markedly increased and negatively correlated with E2 levels in serum [17]. However, the effects of estrogens on the Th17 cell response in the experimental colitis have been scarcely studied.

The present study aimed to investigate the interaction of arctigenin with ER subtype, and identify the critical role that ER plays in arctigenin-mediated inhibition of mTORC1 activation and consequent prevention of Th17 cell differentiation and colitis.

## RESULTS

### Arctigenin inhibits the activation of mTORC1 pathway in T cells by targeting ERβ

**Figure 1:**
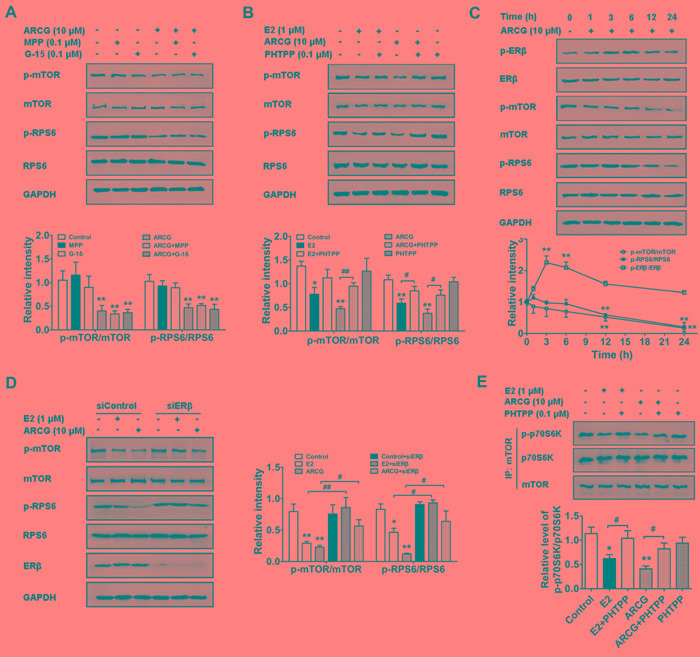
Arctigenin inhibits the activation of mTORC1 pathway in T cells by targeting ERβ **A.** and **B.** Serum-starved EL4 T cells were treated with arctigenin (10 μM) or E2 (1 μM) in the presence or absence of MPP (0.1 μM), PHTPP (0.1 μM) and G15 (0.1 μM) for 24 h. Cells were harvested and lysed, and the levels of p-mTOR, mTOR, p-RPS6, RPS6 and GAPDH were analyzed by immunoblotting. **C.** Serum-starved EL4 cells were treated with arctigenin (10 μM) for 0-24 h, and **D.** EL4 cells were treated with either control siRNA or ERβ siRNA followed by treatment with arctigenin or E2 for 24 h, and the levels of p-mTOR, mTOR, p-RPS6, RPS6 and GAPDH were analyzed by immunoblotting. **E.** Serum-starved Jurkat cells were treated with or without arctigenin (10 μM) or E2 (1 μM) or PHTPP (0.1 μM) for 24 h. mTOR was immunoprecipitated from whole-cell lysates with antibody to mTOR and the immunoprecipitates were used in mTORC1 *in vitro* kinase assay using recombinant p70S6K as a substrate. The kinase assay products were subjected to immunoblotting. Densitometry analysis of immunoblotting was also shown. Data shown are representative of at least three experiments. ^*^P < 0.05, ^**^P < 0.01 *vs*. control group; ^#^*P* < 0.05, ^##^*P* < 0.01 *vs*. indicated group (Dunnett's test). ARCG: arctigenin.

As previously reported, estrogen and its receptors were involved in the regulation of mTORC1 activation [18]. To recognize whether the inhibitory effect of arctigenin on mTORC1 activation was related to ERs, MPP (a selective antagonist of ERα), PHTPP (a selective antagonist of ERβ) and G15 (a selective antagonist of GPER) were added with arctigenin into EL4 cells, respectively. The data showed that arctigenin (10 μM) markedly inhibited the phosphorylation of mTOR and RPS6 in EL4 cells. Neither MPP nor G15 affected the effect of arctigenin, but PHTPP largely diminished the inhibitory effect of arctigenin. As expected, E2 also effectively inhibited the phosphorylations of mTOR and RPS6, which could also be weakened by an addition of PHTPP (Figure 1A and 1B). The findings indicated that ERβ, but not ERα or GPER, mediated the inhibition of arctigenin against mTORC1 pathway in T cells.

To further ascertain the correlation between ERβ activation and mTORC1 inhibition induced by arctigenin in T cells, a time course was observed. As shown in Figure 1C, arctigenin markedly promoted the level of p-ERβ after treatment for 3 hours, and reduced the levels of p-mTOR and p-RPS6 after treatment for 12 hours, suggesting that ERβ activation induced by arctigenin was prior to mTORC1 inhibition. Figure 1D showed that ERβ siRNA transfection almost reversed the inhibition of arctigenin or E2 on the phosphorylations of mTOR and RPS6 in EL4 cells. Consistently, the mTORC1 kinase activity in Jurkat cells was examined by *in vitro* kinase assay using recombinant p70S6K as a substrate. Both arctigenin and E2 markedly reduced the mTORC1 kinase activity in Jurkat cells, as evidenced by the decreased phosphorylation of p70S6K. An addition of PHTPP largely diminished the inhibitory effect of either arctigenin or E2 (Figure 1E). These findings indicated that arctigenin inhibited the activation of mTORC1 pathway in T cells by targeting ERβ.

### Arctigenin displays competition binding ability to ERβ

**Figure 2:**
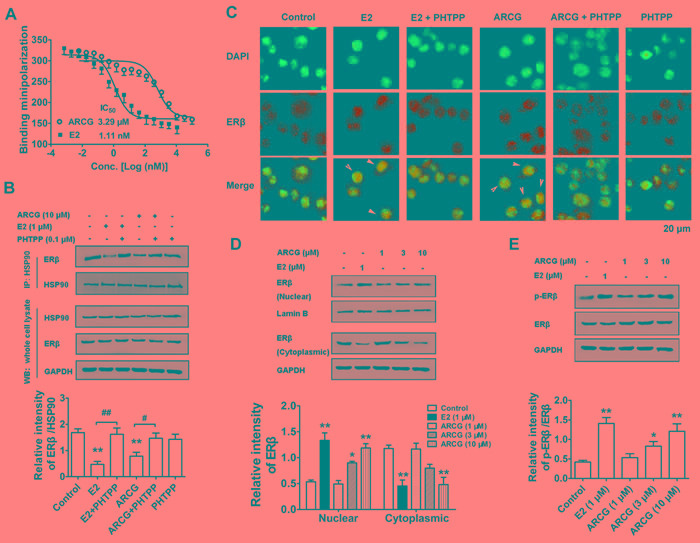
Arctigenin displays binding ability to ERβ and promotes the activation of ERβ in T cells **A.** Purified ERβ was incubated with fluorescent E2 in the absence or presence of different concentrations of E2 and arctigenin for 2 h. EL4 cells were treated with or without arctigenin (10 μM) or E2 (1 μM) or PHTPP (0.1 μM) for 24 h. **B.** Proteins were isolated and immunoprecipitated with antibody against HSP90, the levels of HSP90 and ERβ were analyzed by immunoblotting. **C.** Cells were analyzed by immunofluorescence analysis. Cells were stained with ERβ (green) and then counterstained with DAPI (blue). Scale bar: 20 μm. EL4 cells were treated with arctigenin (1-10 μM) or E2 (1 μM) for 24 h, and **D.** the levels of nuclear and cytoplasmic ERβ or **E.** the levels of p-ERβ and ERβ were evaluated by immunoblotting. Densitometry analysis of immunoblotting was also shown. Results are representative of three independent experiments.^ *^P < 0.05, ^**^P < 0.01 *vs*. control group; ^#^*P* < 0.05, ^##^*P* < 0.01 *vs*. indicated group (Dunnett's test). ARCG: arctigenin.

The above findings indicated that arctigenin could down-regulate mTORC1 activation by activating ERβ in T cells. It was therefore hypothesized that arctigenin might be a ligand of ERβ. To measure the binding affinity of arctigenin to ERβ, competition binding assay for determining the IC_50_ value of arctigenin that bound the full-length ERβ was conducted. Figure 2A showed that arctigenin bound to the ligand-binding domain of ERβ in a concentration-dependent manner with an IC_50_ value of 3.29 μM, and E2 showed an extremely lower value of 1.11 nM, suggesting that arctigenin acted as a moderate ligand for ERβ.

### Arctigenin promotes the activation of ERβ in T cells

The ability of arctigenin to bind to ERβ implied that it might be an ERβ agonist. To recognize whether arctigenin could activate ERβ, its effects on the stability of ERβ/HSP90 complex, and the translocation and phosphorylation of ERβ in EL4 cells were studied. Coimmunoprecipitation test was performed to analyze ERβ/HSP90 complex association in EL4 cells. The results showed that both arctigenin and E2 could facilitate ERβ/HSP90 complex disassociation. PHTPP itself had no obvious effect on the complex disassociation, but largely weakened the effect of arctigenin or E2 (Figure 2B). Both fluorescence and Western blot assays showed that the treatment of arctigenin or E2 induced apparent translocation of ERβ from the cytoplasm to the nucleus, which could be counteracted by the addition of PHTPP (Figure 2C and 2D). Moreover, treatment with arctigenin or E2 markedly promoted the phosphorylation of ERβ in EL4 cells (Figure 2E). Taken together, these results suggested that arctigenin could activate the ERβ in T cells.

### Arctigenin promotes the transcriptional activity of ERβ in T cells

**Figure 3:**
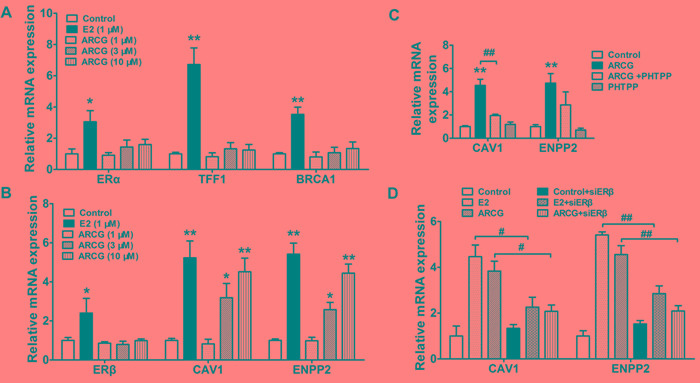
Arctigenin promotes the transcriptional activity of ERβ in T cells EL4 cells were treated with arctigenin (1-10 μM) or E2 (1 μM) for 24 h, and the mRNA expressions of ERα, TFF1, BRCA1 **A.** and ERβ, CAV1 and ENPP2 **B.** were detected by quantitative PCR. EL4 cells were treated with arctigenin (10 μM) or PHTPP (0.1 μM) **C.**, and either control siRNA or ERβ siRNA followed by treatment with arctigenin or E2 **D.** for 24 h, the mRNA expressions of CAV1 and ENPP2 were detected by quantitative PCR. Data are representative of three independent experiments.^ *^P < 0.05, ^**^P < 0.01 *vs*. control group; ^#^*P* < 0.05, ^##^*P* < 0.01 *vs*. indicated group (Dunnett's test). ARCG: arctigenin.

The mRNA expressions of ERα, ERβ and corresponding target genes in EL4 cells treated with or without arctigenin and E2 were measured by qPCR. Figure 3A and 3B showed that arctigenin was lack of noticeable effects on the mRNA expressions of ERα, TFF1, BRCA1 (specific target genes of ERα) and ERβ, whereas it markedly enhanced the mRNA expressions of CAV1 and ENPP2 (specific target genes of ERβ) in a concentration-dependent manner. In contrast, E2 markedly enhanced the mRNA expressions of ERα, ERβ and corresponding target genes. Notably, the enhancement effect of arctigenin on the expressions of CAV1 and ENPP2 in EL4 cells could be largely diminished by either ERβ specific antagonist PHTPP (Figure 3C) or siRNA transfection (Figure 3D). These findings in combination with the affinity to ERβ strongly suggested that arctigenin could function as an agonist for ERβ.

### Arctigenin interferences with the formation of ERβ-raptor-mTOR complex in T cells

**Figure 4:**
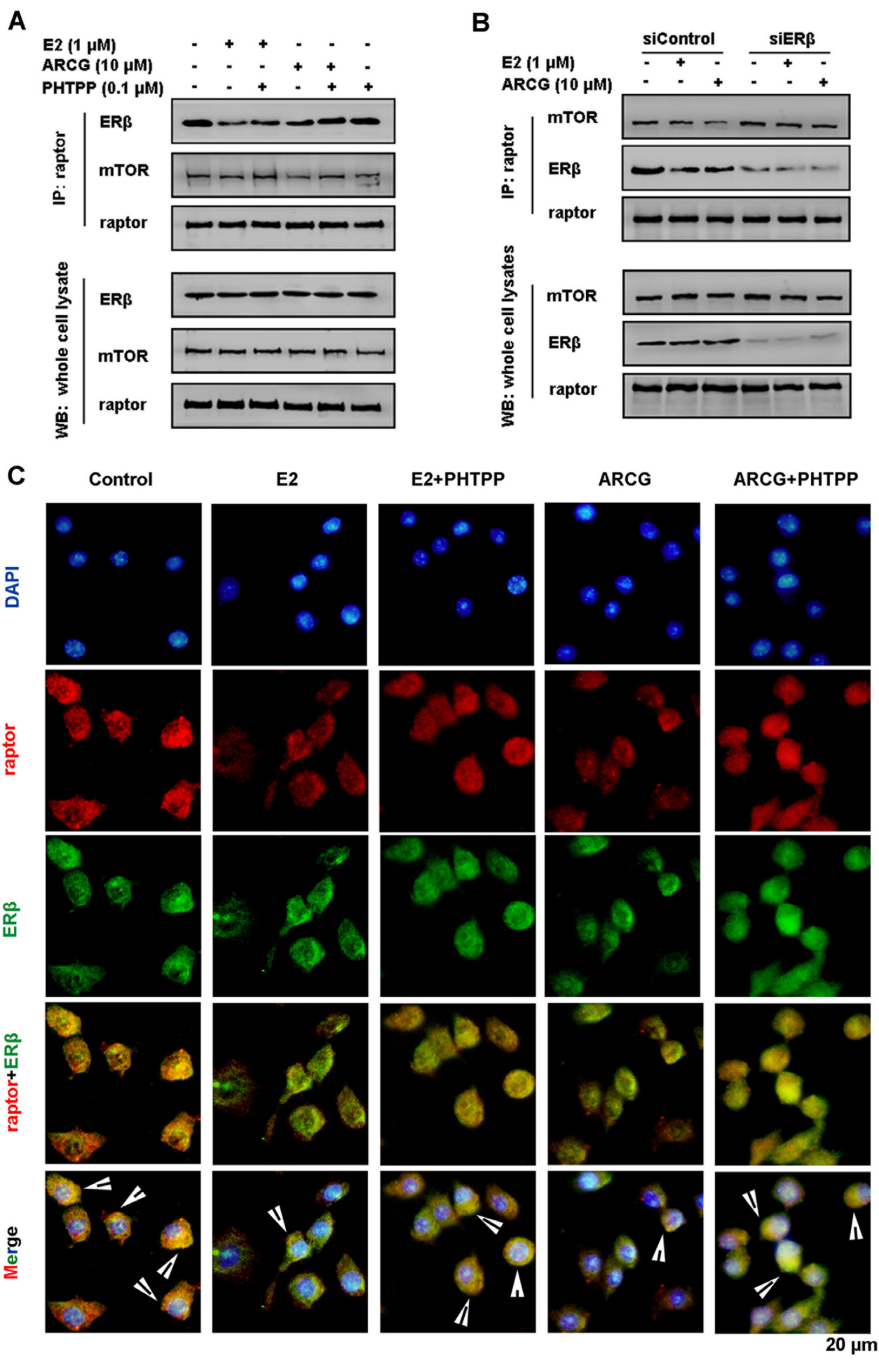
Arctigenin interferences with the formation of ERβ-raptor-mTOR complex in T cells Serum-starved EL4 cells were treated with or without arctigenin (10 μM) or E2 (1 μM) or PHTPP (0.1 μM) **A.**, and either control siRNA or ERβ siRNA followed by treatment with arctigenin or E2 **B.** for 24 h, and proteins were isolated and immunoprecipitated with an antibody against raptor; **C.** Cells were analyzed by immunofluorescence analysis. Cells were co-stained with raptor (red) and ERβ (green) and then counterstained with DAPI (blue). Scale bar: 20 μm. Data shown are representative of three experiments. ARCG: arctigenin.

To determine whether ERβ could directly interact with mTORC1, an antibody against raptor (a critical component of mTORC1) which could pull down its associated proteins was used. As shown in Figure 4A, ERβ and mTOR were markedly co-immunoprecipitated by raptor in EL4 cells. Both arctigenin and E2 were indeed able to decrease the amount of ERβ and mTOR pulled down by the anti-raptor antibody. PHTPP alone had no obvious effect on the amount of ERβ and mTOR, but it markedly reversed the decreased amount of ERβ and mTOR by either arctigenin or E2. Transfection of ERβ siRNA into EL4 cells almost completely reversed the lowered expression of mTOR by arctigenin or E2 (Figure 4B). Immunofluorescence analysis also demonstrated that the formation of raptor and ERβ complex could be interrupted by arctigenin or E2 treatment (Figure 4C). These findings indicated that arctigenin restricted the activation of mTORC1 by interfering with the formation of ERβ-raptor-mTOR complex *via* activating ERβ.

### Arctigenin inhibits the differentiation of Th17 cells through ERβ

**Figure 5:**
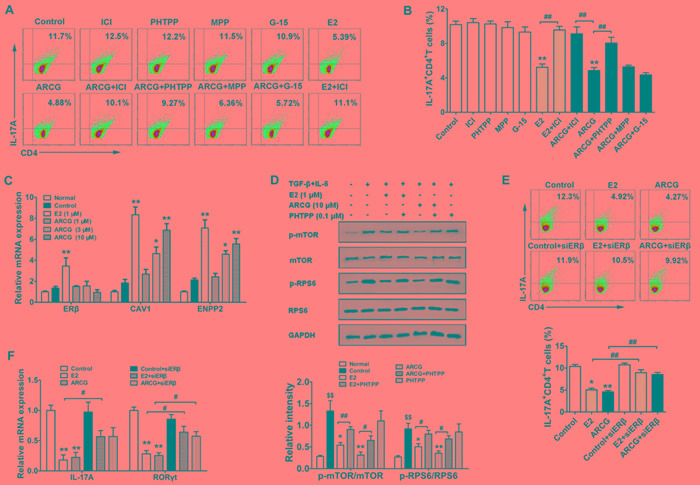
Arctigenin inhibits the differentiation of Th17 cells through ERβ Naïve CD4^+^ T cells were cultured under Th17-polarizing conditions in the presence of arctigenin (10 μM) or E2 (1 μM) with or without MPP (0.1 μM), PHTPP (0.1 μM), G15 (0.1 μM) and ICI (0.1 μM). **A**. and **B**. After treatment for 72 h, IL-17A production in CD4^+^ T cells was assessed by flow cytometry. **C.** After treatment for 48 h, the mRNA expressions of ERβ, CAV1 and ENPP2 were detected by quantitative PCR. **D.** Purified CD4^+^ T cells were pretreated with or without arctigenin (10 μM) or E2 (1 μM) or PHTPP (0.1 μM) for 24 h, followed with Th17 cell polarizing cocktails for 3 h. Cells were harvested and lysed, and the levels of p-mTOR, mTOR, p-RPS6 and RPS6 were analyzed by immunoblotting. **E.** Naïve CD4^+^ T cells were transfected with scrambled siRNA or ERβ siRNA and treated under Th17-polarization condition as indicated. After treatment for 72 h, IL-17A production in CD4^+^ T cells was assessed by flow cytometry. **F.** EL4 cells were treated with either control siRNA or ERβ siRNA followed by treatment with arctigenin or E2 for 24 h, the mRNA expressions of IL-17A and RORγt were detected by quantitative PCR. Results are representative of three independent experiments.^ *^P < 0.05, ^**^P < 0.01 *vs*. control group; ^#^*P* < 0.05, ^##^*P* < 0.01 *vs*. indicated group; ^$$^ *P* < 0.01 *vs*. normal group (Dunnett's test). ARCG: arctigenin.

Previously, we confirmed that arctigenin inhibited the differentiation of Th17 cells and thereby attenuated colitis by restricting the activation of mTORC1 [2]. To define whether ERs play a pivotal role in the inhibition of arctigenin on mTORC1-mediated Th17 cell differentiation, we treated naïve T cells with MPP, PHTPP, G15 and ICI in the presence or absence of arctigenin or E2 under Th17-polarizing cocktails. As shown in Figure 5A and 5B, neither MPP nor G15 showed noticeable effect on the decreased percentage of Th17 (IL-17A^+^CD4^+^ T) cells induced by arctigenin. Both ICI and PHTPP themselves did not affect the Th17 differentiation, but they markedly diminished the inhibitory effect of arctigenin. As expected, E2 could effectively decrease the percentage of Th17 cells. An addition of ICI also diminished the inhibitory effect of E2 on Th17 differentiation. Arctigenin markedly increased the expressions of ERβ target genes CAV1 and ENPP2, but showed no obvious effect on the expression of ERβ during Th17 differentiation (Figure 5C), which coincided well with the fact that arctigenin specifically promoted the expression of ERβ target genes in EL4 cells. Moreover, in primary CD4^+^ T cells stimulated with Th17-polarization cocktails, arctigenin and E2 suppressed the phosphorylations of mTOR and RPS6, and PHTPP treatment markedly diminished the effect of arctigenin and E2 (Figure 5D), which was consistent with the findings obtained in EL4 cells. Transfection of ERβ siRNA into naïve CD4^+^ T cells strikingly abolished the inhibitory effect of arctigenin or E2 on Th17 differentiation (Figure 5E). To further confirm these findings, EL4 cells (constitutive express IL-17A and RORγt) were transfected with either control siRNA or ERβ siRNA followed by treatment with arctigenin or E2. Both arctigenin and E2 suppressed the mRNA expressions of IL-17A and RORγt, whereas ERβ siRNA displayed a markedly blunted response against arctigenin or E2 (Figure 5F). These results indicated that arctigenin inhibited mTORC1-mediated Th17 differentiation dependent on the activation of ERβ rather than the up-regulation of ERβ expression.

### Activation of ERβ induced by arctigenin is associated with inhibition of mTORC1 activation, Th17 response and disease amelioration in DSS-induced colitis mice

**Figure 6:**
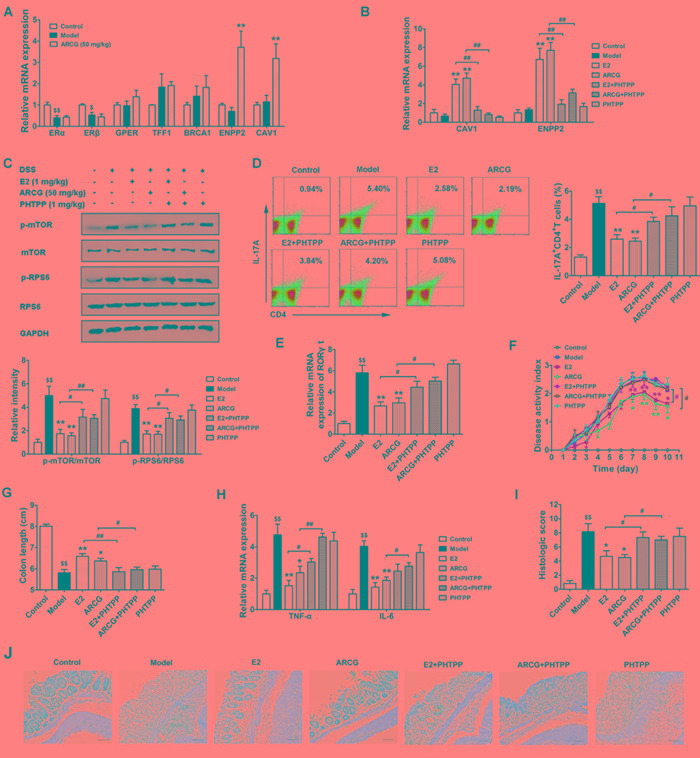
Activation of ERβ induced by arctigenin is associated with inhibition of mTORC1 activation, Th17 response and disease amelioration in DSS-induced colitis mice Mice were treated with 2.5% DSS in drinking water for 7 days, followed by normal drinking water for 3 days to induce colitis. Arctigenin (50 mg/kg) was oral administered daily. PHTPP (1 mg/kg) and E2 (1 mg/kg) were injected intraperitoneal daily. Mice were sacrificed on day 10 after colitis induction. **A.** The mRNA expressions of ERα, TFF1, BRCA1, ERβ, CAV1, ENPP2 and GPER in colons of colitis mice (without OVX) induced by drinking DSS (*n* = 6). **B.** The mRNA expressions of CAV1 and ENPP2 in colons of each group (*n* = 6). **C.** The levels of p-mTOR, mTOR, p-RPS6 and RPS6 in colon tissues were detected by Western blot analysis (*n* = 6). **D.** Intracellular IL-17A production of CD4^+^ T cells in MLNs was measured by flow cytometry, and the percentage of IL-17A^+^CD4^+^ T was evaluated (*n* = 6). **E.** The mRNA expression of RORγt in colons of each group (*n* = 6). **F.** Disease activity index of each group (*n* = 10). **G.** The colon length of each group (*n* = 10). **H.** The mRNA expressions of pro-inflammatory cytokines TNF-α and IL-6 in colons of each group (*n* = 6). **I.** Histological scores of colon from each group (*n* = 6). **J.** Representive histological changes of colon, characterized by distinct infiltration of inflammatory cells and crypt destruction (Magnification × 200). ^$^*P* < 0.05, ^$$^*P* < 0.01 *vs*. control group; ^*^P < 0.05, ^**^P < 0.01 *vs*. model group; ^#^*P* < 0.05, ^##^*P* < 0.01 *vs*. indicated group (Dunnett's test). ARCG: arctigenin.

To ascertain whether arctigenin could activate colonic ERβ, the expressions of ERs and corresponding target genes in the colons obtained from colitis mice (without OVX) were evaluated after treatment of arctigenin for ten days. As shown in Figure 6A, DSS drinking led to a marked decrease of mRNA expression of ERα and ERβ in colons as compared to control group. In line with the *in vitro* findings, arctigenin selectively increased the mRNA expressions of CAV1 and ENPP2, and showed no noticeable effects on the mRNA expressions of GPER, ERβ, ERα, TFF1 and BRCA1.

Furthermore, the correlation between activation of ERβ, consequent inhibition of mTORC1 activation and generation of Th17 cells and eventual anti-colitis effect of arctigenin was verified using ERβ-specific inhibitor PHTPP in OVX-operated mice with colitis induced by drinking DSS. As shown in Figure 6B, PHTPP itself was lack of significant bioactivity. But, it almost completely reversed the expressions of CAV1 and ENPP2 promoted by arctigenin or E2 in colon tissues, which coincided well with our *in vitro* findings. Next, whether the activation of intestinal ERβ by arctigenin could regulate the mTORC1 signaling and consequent Th17 response was explored. The data showed that both arctigenin and E2 markedly decreased the levels of p-mTOR and p-RPS6, the mRNA expression of RORγt in colons and the proportion of Th17 (IL-17A^+^CD4^+^ T) cells in MLNs. PHTPP almost completely diminished the inhibitory effect of arctigenin or E2 (Figure 6C-6E).

The above results indicated that ERβ activation induced by arctigenin played a pivotal role in determining Th17 cell differentiation in both *in vitro* polarization condition and in DSS-induced colitis. To test whether ERβ activation was involved in pathological development of DSS-induced colitis, mice were treated with PHTPP alone or together with arctigenin and E2. The data showed that PHTPP itself did not affect any inflammatory change in the colons, but it markedly inversed the anti-colitis effect of arctigenin or E2. Mice treated with arctigenin or E2 in combination with PHTPP displayed higher DAI scores, reduced colon length (Figure 6F and 6G), increased expressions of pro-inflammatory cytokines TNF-α and IL-6 (Figure 6H) and severe inflammatory damage (Figure 6I and 6J), as compared to those treated with arctigenin or E2 alone. Together, these results indicated that the activation of ERβ induced by arctigenin played a crucial role in inhibition of mTORC1 activation, generation of Th17 cells, and eventual attenuation of pathological changes of colitis, and ERβ agonist might be a promising strategy for prevention of colitis.

## DISCUSSION

The present study mainly dealt with the participation and role of ER in the inhibition of mTORC1 activation by arctigenin. Arctigenin was shown to selectively activate ERβ with a moderate affinity compared with E2. Following the activation of ERβ, arctigenin inhibited the activity of mTORC1, as indicated by disruption of ERβ-raptor-mTOR complex assembly whereby ERβ directly interacted with raptor, and decreased phosphorylation levels of mTOR and RPS6. In addition, the close relationship between arctigenin-mediated ERβ activation and inhibition of mTORC1 activation, Th17 response and amelioration of colitis was verified in DSS-induced colitis mice. The findings substantially increased our understandings of the action mechanism of arctigenin and potential value of ERβ agonists as the therapeutic agents of UC and other Th17-realted diseases.

The mTORC1 plays a critical role in the regulation of various cellular events such as cell growth and proliferation. It can regulate the phosphorylations of the downstream target proteins such as p70S6K and RPS6, and function primarily in the control of translation initiation and nutrient sensing [19]. The activation of mTORC1 signaling is regulated by diverse upstream signals such as positive pathways PI3K/AKT and Ras/ERK, and negative pathways AMPK and PTEN [20]. Notably, a crosstalk between mTORC1 and estrogenic signal pathway has been recently reported [21].

Estrogen, especially E2, acts locally or systemically mainly through three ERs on the target organs and cells, and ERs include ERα, ERβ and GPER. Estrogen not only regulates various physiological process such as cell growth, reproduction and differentiation, but also plays an important role in several pathological processes such as cancer, metabolic and cardiovascular diseases, inflammation and immune diseases [22]. There are reports suggesting that estrogen can activate mTORC1 signaling in breast cancer cells, and conversely, mTORC1 is a crucial activator of ERα transcriptional activity [23]. Moreover, various ERβ agonists play different roles in the regulation of mTOR pathway. The 2, 3-bis (4-hydeoxyphenyl)-propionitrile (DPN) activates mTOR pathway in EAE mice, while liquiritigenin inhibits mTOR activation in glioma cells [8, 24]. ERβ primarily exists in an inactive state in the cytoplasm aggregate bound to HSP90, which is an important molecular chaperone maintaining the stability and function of its client proteins. Upon binding to the ligand, ERβ dissociates from the chaperone protein HSP90 and triggers receptor dimerization, and then ligand-ERβ complex translocate into the nucleus. In the nucleus, ligand-loaded ER binds to estrogen response element (ERE) and regulates gene transcription followed by a range of specific biological activities [25-27]. In this study, ERβ binding assay demonstrated that arctigenin had a moderate affinity to ERβ. To gain insight into the interaction between arctigenin and ERβ, we examined the regulatory effect of arctigenin on ERβ downstream signal pathways and target genes. It was found that arctigenin could promote the dissociation of ERβ with HSP90, increase the phosphorylation of ERβ and facilitate the nuclear translocation of ERβ. Further studies confirmed that arctigenin could increase the mRNA expressions of CAV1 and ENPP2 in an ERβ-dependent manner. These results confirm that arctigenin is an agonist of ERβ.

It has been reported that ERβ can regulate mTOR activation *via* multiple mechanisms, such as increasing PTEN expression and decreasing phosphorylation of AKT [18, 28]. In this study, we found that the activation of ERβ could decrease mTORC1 activation. E2 and arctigenin decreased the phosphorylations of mTOR and RPS6 in EL4 and primary CD4^+^ T cells, which were diminished by an addition of either PHTPP or ERβ siRNA. The decrement effect of E2 on the phosphorylation of mTOR in lymphocytes was consistent with a previous report that E2 inhibited mTOR activation in osteoblasts [7]. As reported previously, phytoestrogen calycosin and ERβ agonist liquiritigenin could up-regulate ERβ expression and inhibit the activation of mTOR pathway in breast cancer cells and glioma cells, respectively [8, 9]. In contrast, arctigenin decreased mTORC1 activation through the activation of ERβ rather than the up-regulation of ERβ expression. Antagonism of ERβ abolished the inhibitory effect of arctigenin on the kinase activity of mTORC1 in Jurkat cells. In consistent with this concept, activation of ERβ signaling in EL4 cells by exposure to E2 markedly facilitated the inhibitory effect of arctigenin on the phosphorylation of RPS6. Coimmunoprecipitation of ERβ with raptor indicated a direct binding effect between ERβ and mTORC1. Arctigenin or E2 decreased the stability of ERβ-raptor-mTOR complex. Deletion of ERβ or exposure to PHTPP largely counteracted the effect of arctigenin or E2. Thus, ERβ was thought to be the key mediator for the inhibitory effect of arctigenin on the mTORC1 activation.

ER agonists have been previously documented to regulate the differentiation of CD4^+^ T cell *in vitro* [29-31]. Moreover, estrogen deficiency was shown to facilitate the differentiation of Th17 cells *in vivo* [32]. RORγt promoter region possesses ERE binding site, and E2 can recruit REA *via* ERα to the EREs on the RORγt promoter region, thus inhibit RORγt expression and Th17 differentiation [33]. In this study, we assessed the participation of ER subtypes in arctigenin-mediated inhibition of Th17 cell differentiation. To achieve this goal, specific inhibitors (such as MPP, PHTPP and G-15) and ERβ siRNA were used in the presence or absence of arctigenin under Th17 cell differentiation condition, and the data showed that only deficiency of ERβ could largely diminish the inhibitory effect of arctigenin. Moreover, silencing of ERβ in EL4 cells also counteracted the lowered mRNA expressions of IL-17A and RORγt induced by arctigenin.

ERβ has been shown to be beneficial in experimental animal model of UC [34]. In active UC patients, ERβ expression markedly decreased at both mucosal and systemic levels [35]. In line with this, we found that ERβ expression largely reduced in the colons obtained from DSS-induced colitis mice. The mice lacking functional ERβ showed aggravated prognosis of colitis compared to wild-type littermates and ERα knockout mice [34]. Furthermore, treatment with a specific agonist of ERβ could decrease disease severity in colitis mice [14]. Consistently, we found that arctigenin and E2 exerted anti-colitis efficacy by ways dependent on ERβ.

In our previous studies [3], arctigenin was demonstrated to exert anti-inflammatory efficacy *via* down-regulating the activation of MAPKs and NF-κB pathways in the inflamed colon tissues. There were a lot of reports suggesting that the ERβ agonists could inhibit the activation of MAPKs and NF-κB pathways. E2 could decrease the phosphorylations of ERK and p38 MAPK in liver tissues and adipocytes, respectively. It also inhibited the translocation of NF-κB p65 to nucleus in adipocytes [36, 37]. Whether and how MAPKs and NF-κB pathways involve in the arctigenin-induced inhibition of abnormal Th17 cell response in colitis need to be identified.

In conclusion, this study identified that arctigenin is a selective ERβ agonist. It activates ERβ, and therefore inhibits mTORC1 activation and destroys mTOR-raptor interaction through binding to raptor, thus leading to a down-regulation of Th17 cell differentiation and amelioration of colitis. The findings also suggest that ERβ might be a potential therapeutic target for Th17 cells-related diseases.

## MATERIALS AND METHODS

### Reagents

Arctigenin (C_21_H_24_O_6_, MW: 372.41, purity = 99.9%) was purchased from Zelang Pharmaceutical Technology Co., Ltd. (Nanjing, China). ICI-182780 (ICI, ER non-specific antagonist) was kindly presented by Abmole Bioscience (Kowloon, Hong Kong). E2 was purchased from Sigma (St. Louis, MO, USA). Methylpiperidino pyrazole (MPP, ERα selective antagonist), 2-phenyl-3-(4-hydroxyphenyl)-5, 7-bis (trifluoromethyl)-pyrazolo [1, 5-alpha] pyrimidine (PHTPP, ERβ selective antagonist) and G-15 (G protein coupled ER (GPER) selective antagonist) were purchased from Cayman Chemical (Ann Arbor, MI, USA). *In vitro* study, arctigenin, E2, ICI, MPP, PHTPP and G-15 were dissolved in DMSO, stored at -20 °C, and further diluted in cell culture medium so that the final DMSO concentration did not exceed 0.1% (v/v). *In vivo* study, arctigenin, E2 and PHTPP were dissolved in olive oil. FITC-anti-CD4, APC-anti-IL-17A and cell stimulation cocktail (containing phorbol 12-myristate 13-acetate (PMA), ionomycin, brefeldin A and monensin) were purchased from eBioscience (San Diego, CA, USA). Recombinant murine IL-6 and human TGF-β1 (R&D Systems, Minneapolis, MN, USA) were used. Purified anti-mouse CD3e, anti-mouse CD28, anti-mouse IL-4 and anti-mouse IFN-γ mAbs were purchased from BD Pharmingen (San Diego, CA, USA). HiScript RT SuperMix and Ace qPCR SYBR Green Master Mix were purchased from Vazyme (Nanjing, China). TRIzol reagent was procured from Invitrogen (Carlsbad, CA, USA). Antibodies against p-mTOR, p-p70S6K, p70S6K, p-ERβ, ERβ, HSP90, Rhodamine-IgG and FITC-IgG as well as protein A/G agarose beads were purchased from Bioworld Technology, Inc. (Georgia, USA). Antibodies against p-RPS6 and RPS6 (Sangon Biotech, Shanghai, China), and against mTOR (Abcam, Cambridge, UK) as well as against regulatory-associated protein of mTOR (raptor) (Proteintech, Wuhan, China) were used. Other chemical products used were of the analytical grade available.

### Animals

Female C57BL/6 mice (18-20 g), 6 to 8 week-old, were purchased from the Comparative Medicine Center of Yangzhou University (Yangzhou, China), and allowed to acclimate for one week. They were fed with a standard chow diet and water *ad libitum* with a 12 h light/dark cycle. All animal experiments were strictly performed in accordance with the Guide for the Care and Use of Laboratory Animals and the related ethical regulations of China Pharmaceutical University. All efforts were made to minimize animal suffering and to reduce the number of animals used in the study.

### Ovariectomy, colitis induction and treatment in mice

Mouse ovariectomy (OVX) was performed as previously reported [34, 38]. Mice were anesthetized with chloral hydrate, clipped dorsally, and aseptically scrubbed. A single longitudinal skin incision was made on the dorsal midline. The skin was then manipulated over the fat pad where the ovarium resides. A small cut was made in the fat pad, and the ovarium was found and the bilateral ovaries were removed.

After one week of recovery period for complete depletion of endogenous sex hormones, OVX-operated mice were treated with DSS to induce colitis. Mice were given drinking water containing 2.5% (w/v) DSS *ad libitum* for consecutive seven days and then replaced by normal drinking water for further three days. For treatments, arctigenin (50 mg/kg) was orally administered daily for ten days. PHTPP (1 mg/kg) and E2 (1 mg/kg) were intraperitoneally injected daily for ten days. Mice in the control and model groups received an equal volume of olive oil. Body weight, stool consistency and gross blood were observed every day. The disease activity index (DAI) was calculated as previously described [3]. On day ten, mice were sacrificed, and the colons and mesenteric lymph nodes (MLNs) were collected.

### Histological evaluation

For histological evaluation, formalin-fixed colon tissues were embedded in paraffin. sections (5 μm) were stained with hematoxylin and eosin. Histological scoring was performed by a blinded pathologist as described previously [3].

### Cell culture

T cells from lymph nodes and spleens of normal C57BL/6 mice, mouse lymphoma EL4 T cells and human lymphoma Jurkat T cells (ATCC, Manassas, VA, USA) were maintained in RPMI 1640 medium (Gibco, Carlsbad, CA) supplemented with 100 U/mL of streptomycin, 100 U/mL of penicillin and 10% fetal calf serum (Biological Industries, Israel) under a humidified 5% (v/v) CO_2_ atmosphere at 37 °C.

### Th17 differentiation and flow cytometry

Naïve CD4^+^ T cells from lymph nodes and spleens of 6 to 8 week-old mice were purified with magnetic beads (CD4^+^CD62L^+^ T Cell Isolation Kit II, Miltenyi Biotech), and the purity of isolated T cells routinely exceeded 95%. Naïve CD4^+^ T cells were stimulated with Th17 polarization medium consisting of anti-CD3 and anti-CD28 (2 μg/mL), rhTGF-β1 (2 ng/mL), rIL-6 (40 ng/mL), anti-IL-4 (10 μg/mL) and anti-IFN-γ (10 μg/mL) antibodies. As indicated, the cells were simultaneously treated with or without arctigenin (10 μM), PHTPP (0.1 μM), MPP (0.1 μM), G-15 (0.1 μM), ICI (0.1 μM) and E2 (1 μM). After treatment for three days, the cells were harvested for analysis. The frequencies of Th17 cells were analyzed by flow cytometry as previously described [2].

### Quantitative real-time PCR analysis

Total RNA from colon tissues of colitis mice or cell pellets was isolated using TRIzol reagent according to the manufacturer's instructions. Quantitative PCR was performed as previously described [2]. The primer sequences in the reaction used were listed in Table 1. All values were expressed relative to the expression of GAPDH.

**Table 1:** Mouse primer pairs used in real-time PCR

Gene		Sequence (5′ - 3′)	Length (bp)	Annealing temperature (°C)	
GAPDH	forward	GGTGAAGGTCGGTGTGAACG	233		55
	reverse	CTCGCTCCTGGAAGATGGTG			
ERα	forward	TCC AGC AGT AAC GAG AAA GGA	88		57
	reverse	AGC CAG AGG CAT AGT CAT TGC			
ERβ	forward	CTG TGC CTC TTC TCA CAA GGA	129		57
	reverse	TGC TCC AAG GGT AGG ATG GAC			
GPER	forward	ATG GAT GCG ACT ACT CCA GC	179		57
	reverse	AAG AGG GCA ATC ACG TAC TGC			
TFF1	forward	AGC ACA AGG TGA TCT GTG TCC	135		57
	reverse	GGA AGC CAC AAT TTA TCC TCT CC			
BRCA1	forward	CGA ATC TGA GTC CCC TAA AGA GC	89		57
	reverse	AAG CAA CTT GAC CTT GGG GTA			
CAV1	forward	ATG TCT GGG GGC AAA TAC GTG	132		57
	reverse	CGC GTC ATA CAC TTG CTT CT			
ENPP2	forward	TTT GCA CTA TGC CAA CAA TCG G	213		57
	reverse	GGA GGC ACT TTA GTC CTG TAC TT			

### Western blot analysis

Cytoplasmic and nuclear protein extracts were prepared by Cytoplasmic and Nuclear Protein Extraction Kits according to the manufacturer's recommendations (Vazyme, Nanjing, China). The cytoplasmic, nuclear extracts and whole cell lysates were prepared and subjected to Western blot analysis as described previously [2]. Bands were detected immunologically using antibodies against ERβ (1:800), p-ERβ (1:800), HSP90 (1:1000), p-mTOR (1:1000), mTOR (1:2000), p-p70S6K (1:800), p70S6K (1:800), p-RPS6 (1:1000) and RPS6 (1:1000), respectively. All blots were stripped and incubated with GAPDH (1:5000) or Lamin B (1:1000) antibody to ascertain equal loading of the proteins.

### Immunofluorescence assay

The immunofluorescence assays for ERβ and raptor were performed in accordance with a routine procedure. The EL4 cells were fixed in 4% paraformaldehyde, permeabilized with 1% Triton X-100 for 15 min and blocked with 3% BSA for 1 h. Cells were incubated with antibody for either ERβ (1:100) or raptor (1:100) overnight at 4 °C and then with FITC-conjugated IgG (1:100) or Rhodamine-conjugated IgG (1:100) for 1 h. Cells were counter-stained with DAPI for 5 min, and observed under a fluorescence microscope (Olympus, Tokyo, Japan).

### Coimmunoprecipitation assay

EL4 or Jurkat T cells were collected and lysed in ice-cold NP40 lysis buffer (Beyotime, Shanghai) for 20 min and centrifuged at 10,000 g for 10 min at 4 °C. For HSP90, raptor and mTOR coimmunoprecipitation experiments, the supernatant fractions were incubated overnight at 4 °C with 2 μg of rabbit anti-HSP90, anti-raptor and anti-mTOR antibody, respectively, and precipitated with protein A/G agarose beads for additional 4 h at 4 °C. The beads were washed with NP40 lysis buffer for four times. The immunoprecipitated proteins were separated by SDS-PAGE, and Western blot was performed with the indicated antibodies.

### mTOR activity assay

*In vitro*, the mTOR activity assay in Jurkat T cells was performed as we previously reported [2].

### ERβ competition binding assay

The PolarScreen ERβ Competitor Assay kit (Invitrogen life Technologies, Carlsbad, CA) was used to determine the IC_50_ values of compounds that bind the full-length ERβ. According to the manufacturer's instructions, we can test whether arctigenin is a ligand for ERβ using this kit. In this experiment, arctigenin and E2 were serially diluted from 0.01 nM to 100 μM, and mixed well with reagent solution (23 nM full-length ERβ and 4.5 nM Fluormone Tracer premixed solution) in a 384-well plate. The E2 was used as a positive control. After incubation for 2 h in the dark to protect the reagents from light, the fluorescence polarization of each well was read in a fluorescence polarization plate reader (excitation = 485 nm, and emission = 535 nm). The concentration of the test compound that resulted in a half-maximal shift in polarization value equaled to the IC_50_ value, which was a measure of the relative affinity of the test compound for ERβ ligand binding domain. Data were analyzed from three independent experiments. The IC_50_ values were calculated by Graph pad Prism 5 software.

### Transient transfection

The ERβ (with target sequence GGTCCTGTGAAGGATGTAA) and scrambled siRNA were synthesized by RiboBio Co. (Guangzhou, China). EL4 and naïve CD4^+^ T cells were transfected with the siRNA using Lipofectamine 2000 (Invitrogen, Carlsbad, CA) according to the manufacturer's instructions. Silence efficiency was assessed by quantitative PCR analysis after transfection for 24 h. The cells were then prepared for further analysis.

### Statistical analysis

All values were expressed as means ± standard errors of the means (S.E.M.). Data sets were performed using one way analysis of variance (ANOVA) followed by Dunnett's test between control and experimental groups. Values of *P* < 0.05 were considered significant.
